# Patient reactions to a web-based cardiovascular risk calculator in type 2 diabetes: a qualitative study in primary care

**DOI:** 10.3399/bjgp15X683953

**Published:** 2015-03-02

**Authors:** Tom Nolan, Charlotte Dack, Kingshuk Pal, Jamie Ross, Fiona A Stevenson, Richard Peacock, Mike Pearson, David Spiegelhalter, Michael Sweeting, Elizabeth Murray

**Affiliations:** Brockwell Park Surgery, London.; Psychology Department, University of Bath, Bath.; e-Health Unit, Research Department of Primary Care and Population Health, University College London, London.; e-Health Unit, Research Department of Primary Care and Population Health, University College London, London.; e-Health Unit, Research Department of Primary Care and Population Health, University College London, London.; Whittington Health Library, The Whittington Hospital, London, UK.; Statistical Laboratory, Centre for Mathematical Sciences;; Statistical Laboratory, Centre for Mathematical Sciences;; Department of Public Health and Primary Care, Strangeways Research Laboratory, University of Cambridge, Cambridge.; e-Health Unit, Research Department of Primary Care and Population Health, University College London, London.

**Keywords:** diabetes mellitus, type 2, patients, primary care, qualitative research, risk assessment

## Abstract

**Background:**

Use of risk calculators for specific diseases is increasing, with an underlying assumption that they promote risk reduction as users become better informed and motivated to take preventive action. Empirical data to support this are, however, sparse and contradictory.

**Aim:**

To explore user reactions to a cardiovascular risk calculator for people with type 2 diabetes. Objectives were to identify cognitive and emotional reactions to the presentation of risk, with a view to understanding whether and how such a calculator could help motivate users to adopt healthier behaviours and/or improve adherence to medication.

**Design and setting:**

Qualitative study combining data from focus groups and individual user experience. Adults with type 2 diabetes were recruited through website advertisements and posters displayed at local GP practices and diabetes groups.

**Method:**

Participants used a risk calculator that provided individualised estimates of cardiovascular risk. Estimates were based on UK Prospective Diabetes Study (UKPDS) data, supplemented with data from trials and systematic reviews. Risk information was presented using natural frequencies, visual displays, and a range of formats. Data were recorded and transcribed, then analysed by a multidisciplinary group.

**Results:**

Thirty-six participants contributed data. Users demonstrated a range of complex cognitive and emotional responses, which might explain the lack of change in health behaviours demonstrated in the literature.

**Conclusion:**

Cardiovascular risk calculators for people with diabetes may best be used in conjunction with health professionals who can guide the user through the calculator and help them use the resulting risk information as a source of motivation and encouragement.

## INTRODUCTION

Risk calculators are available to clinicians and the public to estimate the risk of developing an array of illnesses including cardiovascular disease, osteoporosis, diabetes, and many types of cancer.^1^–^3^ Cardiovascular risk estimates have been incorporated into NHS Health Checks and National Institute for Health and Care Excellence guidelines for hypertension and hyperlipidaemia,^4^,^5^ and the American Heart Association suggest that all adults aged >40 years should know their global cardiovascular risk.^6^

The assumption underlying promotion of risk calculators is that improving the accuracy of perception of risk will lead to adoption of appropriate interventions to reduce risk, such as improved health behaviours or greater adherence to preventive medications.^7^,^8^ This assumption is related to the Health Belief Model, which states that individuals are likely to change a behaviour when they perceive a personal threat or illness as resulting from that behaviour and believe changing that behaviour will effectively avert the threat.^9^ Accurate personalised information in a risk calculator could impact on the user’s perceived susceptibility and hence perceived threat. A risk calculator allowing a user to see the benefits of changing one or more risk factors could impact on the perceived benefits of change, while use of the risk calculator could itself be a cue to action.

Before a risk calculator can have these potential impacts, users first need to understand and make sense of the information in the risk calculator, and accept that the information is personally relevant. This sense of personal relevance includes agreeing that the ‘disease X’ is potentially serious and worth avoiding. Second, users have to be convinced that the benefits the calculator suggests can be achieved, in terms of avoiding ‘disease X’, are significant, and worth the effort required to achieve change. Unwanted impacts are also possible. Users could be demotivated if they felt that the benefits achieved by change were not worth the effort, made increasingly anxious or depressed about their health, or feel disempowered about their ability to influence health outcomes.^10^,^11^

The empirical data on use of risk calculators by patients are sparse and unconvincing. Randomised controlled trials suggest that although presenting risk to patients can have a small impact on the accuracy of risk perception and on intention to change, there is little or no impact on behavioural outcomes or overall risk.^12^,^13^ In view of this uncertainty, this study was conducted to explore user reactions to a cardiovascular risk calculator for people with type 2 diabetes. The study aimed at identifying users’ cognitive and emotional reactions to the presentation of risk, with a view to understanding whether and how such a calculator could help motivate patients with type 2 diabetes to adopt healthier behaviours and/or improve adherence to medication.

**How this fits in**

Risk calculators are increasingly used to estimate individual patient’s cardiovascular risk and guide management. It has been argued that providing patients with individualised calculations of global cardiovascular risk could promote healthy behaviours and improved self-management; however, trial data do not support this hypothesis. This study explores the responses of patients with type 2 diabetes to an interactive, personalised cardiovascular risk calculator. Awareness of these responses may help GPs and practice nurses who wish to use risk calculators as a motivational intervention in consultations.

## METHOD

### Design

This was a qualitative study combining data from focus groups and individual user experience, ‘think-aloud’, and semi-structured interviews.

### Participants and recruitment

Adults with type 2 diabetes were recruited through advertisements on the Diabetes UK website (www.diabetes.org.uk; the UK’s leading diabetes charity), black, minority and ethnic health forum (bmehf.org.uk), local council websites, and posters displayed at local GP practices and diabetes groups. Responders were posted information about the study, a consent form, and a screening questionnaire that was used to check eligibility and recruit participants who varied across sex, age, years since diagnosis, diabetes medication, and experience of using the internet.

### Intervention

The risk calculator was developed to form part of a web-based self-management programme for people with type 2 diabetes, called Healthy Living for People with Diabetes (HeLP-Diabetes). This programme was developed by a multidisciplinary team with substantive user input,^14^ and addressed the three tasks of self-management described by Corbin and Strauss, namely medical management, emotional management, and role management.^15^ The risk calculator was developed at the University of Cambridge by adapting the statistical approach and computer algorithms developed for a cardiovascular risk calculator for people without diabetes.^16^ Risk estimates were based on the UK Prospective Diabetes Study (UKPDS) data.^17^,^18^ In response to data from focus groups held during development, the UKPDS data were supplemented with data from Cochrane or Health Technology Assessment systematic reviews or large randomised controlled trials that reported the effects of medication and behavioural modification on cardiovascular disease outcomes (rates of myocardial infarction and stroke) and mortality rates.

**Figure 1.**
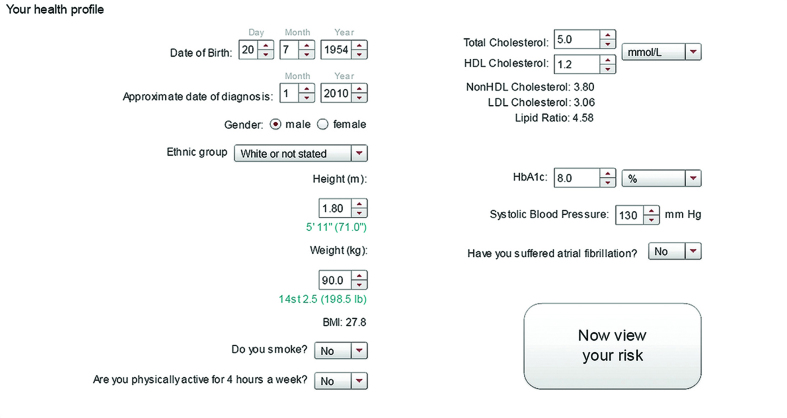
***Information entry page in risk calculator. This example shows a 60-year-old white male diagnosed with diabetes 5 years ago, who is a non-smoker, does less than 4 hours of physical activity a week, and has the following values: weight 90 kg, height 1.80 m, HbA1c 64 mmol/mol (8%), total cholesterol 5 mmol/L, HDL 1.2 mmol/L, systolic blood pressure 130 mmHg. These parameters are carried forward in Figure 2, which also demonstrates the effect on risk profile for this man if he increased his physical activity levels to more than 4 hours/week.***

For the think-aloud and qualitative interviews, users were asked to read a brief introductory page and then to provide information about themselves and their health; including their age, sex, ethnic group, smoking status, level of physical activity, height, weight, date of diagnosis of diabetes, glycated haemoglobin (HbA1c), lipids (total and high density lipoprotein [HDL] cholesterol), history of atrial fibrillation, and systolic blood pressure values (Figure 1). Participants were asked to obtain these clinical data in advance from their general practice.

**Figure 2.**
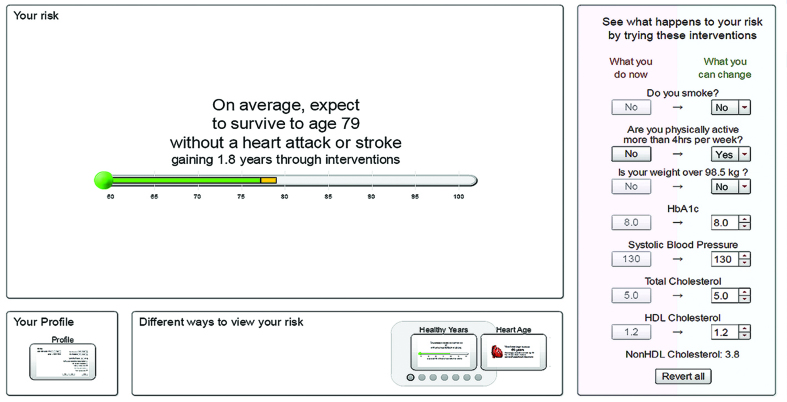
***The Healthy Years format shows the average age of survival without a heart attack or stroke based on the user’s profile. In this example the user has gained 1.8 years by selecting the intervention (on the right of the screen) to become physically active for more than 4 hours per week.***

**Figure 3.**
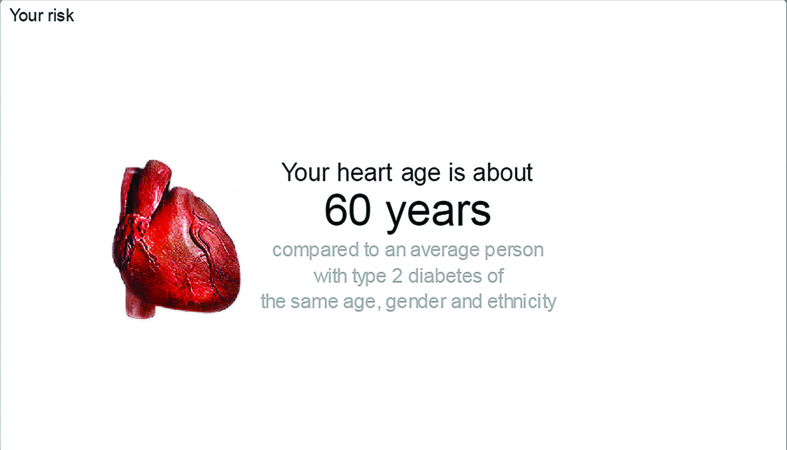
***Heart age compares the user’s heart age with an average person with type 2 diabetes of the same age, sex, and ethnic group.***

**Figure 4.**
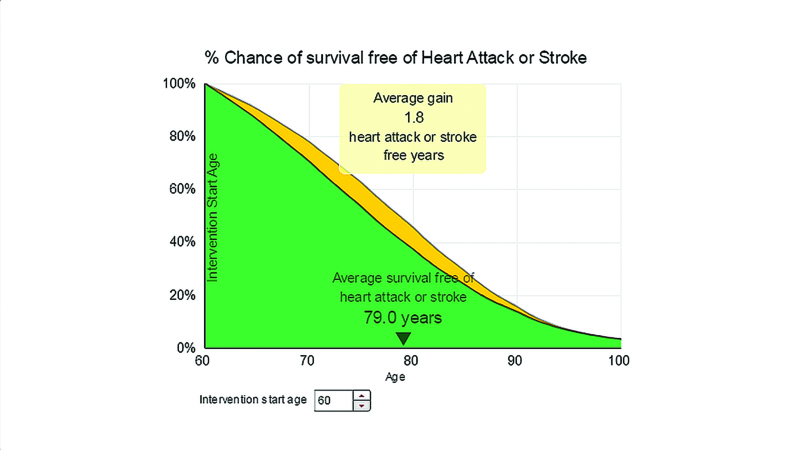
***Outlook plots age against percentage chance of survival free of heart attack or stroke. In the example the yellow area indicates the expected heart attack or stroke-free life-years gained when the user selected the intervention to become physically active for more than 4 hours per week.***

**Figure 5.**
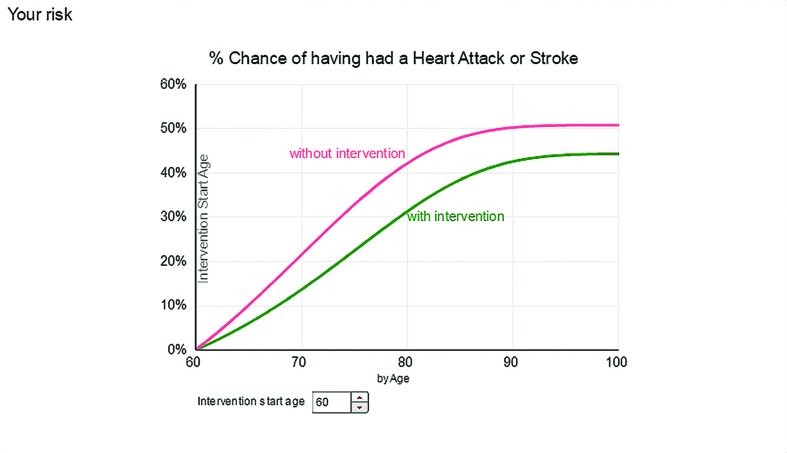
***Risk By Age plots age against risk of having a heart attack or stroke. In this example the risk of having had a heart attack or stroke by the age of 80 is over 40%, dropping to 30% with immediate (at age 60) and continued initiation of physical activity.***

**Figure 6.**
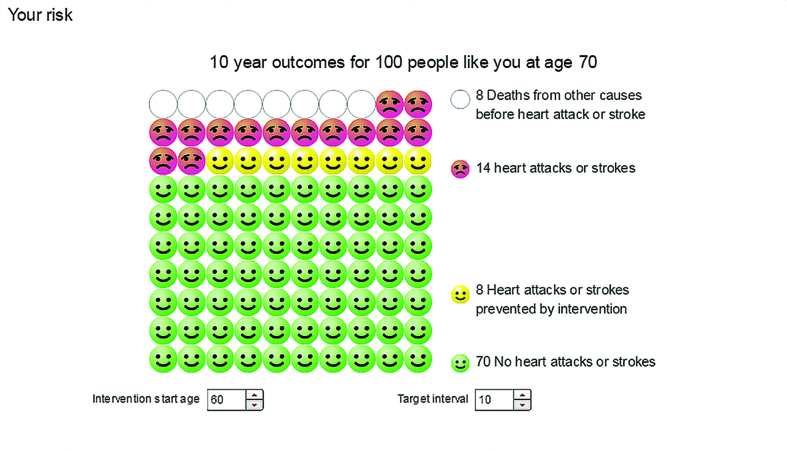
***Outcome shows 10-year outcomes for ‘100 hundred people like you’ (the time horizon can be changed by the user). Green smiling faces represent people who survive and do not have a heart attack or stroke within 10 years; yellow smiling faces represent heart attacks or strokes prevented by the intervention (in this example, physical activity); red unhappy faces represent people who have had a heart attack or stroke. Blank circles represent deaths from other causes. Note that this format includes death from other causes in addition to heart attack and stroke risk, whereas Risk By Age (Figure 5) shows only heart attack and stroke risk.***

**Figure 7.**
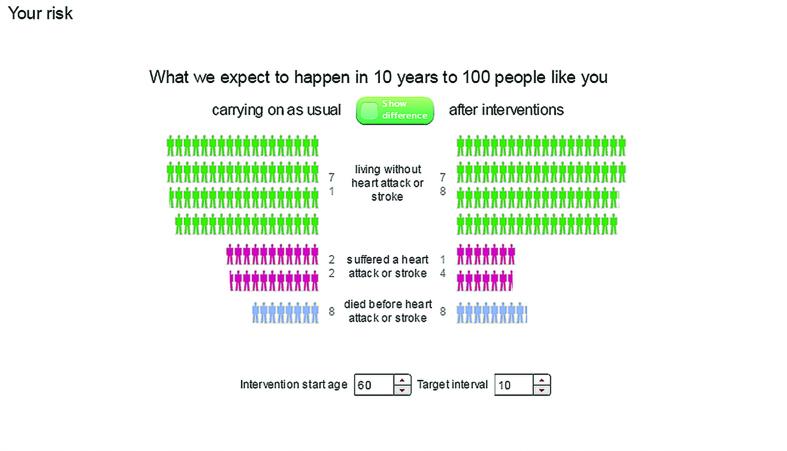
***Balance shows what is expected to happen to ‘100 people like you’ in 10 years who either carry on as usual (on the left) or who make the interventions (on the right) selected in the interventions panel (see Figure 2). Green figures represent people who survive and do not have a heart attack or stroke within 10 years, pink figures represent people who have a heart attack or stroke, and blue figures represent people who die of other causes.***

**Figure 8.**
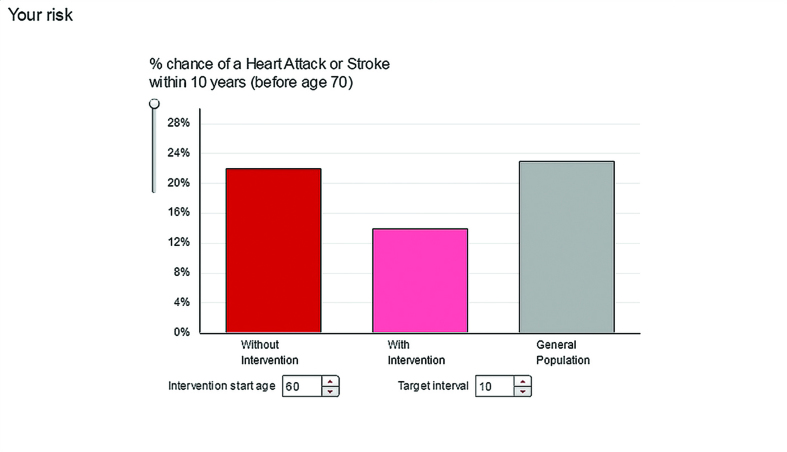
***Shows the percentage chance of having a heart attack or stroke within 10 years, without (red bar) and with (pink bar) selected interventions and compared with other people with diabetes (grey bar).***

The calculator provided personalised estimates of risk of heart attack and stroke, following best practice in presenting risk information by presenting risk in numbers, words, and graphically, using absolute and relative risk, using natural frequencies, and presenting the information in a range of formats.^19^–^24^ Seven different visual presentations of risk were included (Figures 2–8).

Once participants had looked at their current risk and indicated to the interviewer that they had understood this information, they were encouraged to alter the entries for their modifiable parameters (physical activity, smoking, weight, HbA1c, lipid levels, and systolic blood pressure) to explore the impact on their future cardiovascular risk. Figures 2–8 show the different visual presentations of risk that would be calculated for a 60-year-old white male with the parameters entered in Figure 1.

### Data collection

Data collection took place in three waves. First, focus groups were held to explore participants’ overall views about including a cardiovascular risk calculator in the self-management programme and to inform the initial development of the risk calculator.

Second, usability testing^23^ was carried out to optimise the overall navigation and presentation of risk information. Testing with five users is thought to be optimally cost-effective^25^ as the method quickly identifies areas in which navigation or page layout need improving.

Third, after revising the calculator in line with results from user testing, naïve users were recruited to undertake think-aloud interviews as they used the calculator, followed by semi-structured interviews with a topic guide that explored users’ experiences and reactions. The think-aloud interviews were carried out by a GP academic trainee. Data collection in this third wave continued until theoretical saturation was reached.

Focus groups and interviews were audiotaped and transcribed verbatim with transcripts validated against the original tapes. Additional field notes from the individual interviews were retained.

### Data analysis

Data were analysed iteratively with data collection, so that subsequent focus groups or interviews could explore early emergent themes. Data were analysed by a multidisciplinary group, including experienced qualitative researchers with backgrounds in general practice, health psychology, and sociology, and expertise in e-health.

Analytical methods were selected according to the purpose of each wave of data collection. Thus, focus group data were analysed using a framework approach, with an emphasis on obtaining practically useful information to help the team decide whether to pursue the development of a risk calculator, which modifiable risk factors should ideally be included in the risk calculator (although not all parameters requested by users could be included as high quality cohort or RCT data were not available), and how the risk information should be presented. Usability testing informed revision of the navigation and layout of the risk calculator and outputs. Finally, the think-aloud and semi-structured interviews were subjected to thematic analysis. Transcripts were read and re-read by three investigators, with selected transcripts read by three additional investigators. Emergent themes were discussed in data clinics, and transcripts were coded. The coding framework from these interviews was then applied to the focus group transcripts and usability testing. Every effort was made to identify disconfirming data that did not fit with emergent themes. The final analysis was agreed by the six investigators who read the transcripts.

Illustrative quotes in the results are identified by initial (FG = focus group, UT = user testing, II = individual interviews) followed by a number and key demographic information. IV refers to the interviewer.

## RESULTS

Thirty-six participants contributed data; 15 in two focus groups, five undertook usability testing, with a further 16 think-aloud and semi-structured interviews. Demographic information is presented in Table 1.

**Table 1.** Demographic characteristics of participants

	**Focus groups (*n*= 15)**	**Usability testing (*n*= 5)**	**Think-aloud and semi-structured interviews (*n*= 16)**
**Sex**			
Male, *n* (%)	8 (53)	2 (40)	10 (63)
Female, *n* (%)	7 (47)	3 (60)	6 (37)

**Age, years**			
Mean (SD)	61.7 (7.1)	59.2 (8.9)	61.9 (8.8)
Range	51–73	49–70	44–77

**Ethnic group**			
White British, *n* (%)	11 (73)	3 (60)	15 (94)
White Irish, *n* (%)	1 (7)	0 (0)	0 (0)
Black, *n* (%)	3 (20)	1 (20)	0 (0)
Other, *n* (%)	0 (0)	1 (20)	1 (6)

**Time since diagnosis, years**			
Mean (SD), years	10.1 (8.5)	2.6 (2.1)	5.1 (7.6)
Range	3 months–36 years	2 months–5 years	1–31 years

**Diabetes treatment**			
Lifestyle only, *n* (%)	4 (27)	3 (60)	3 (19)
Lifestyle + oral medication, *n* (%)	8 (53)	2 (40)	12 (75)
Lifestyle + oral medication + insulin or other injectable, *n* (%)	3 (20)	0 (0)	1 (6)

**Self-defined internet experience**			
Novice, *n* (%)	0 (0)	0 (0)	0 (0)
Basic, *n* (%)	1 (7)	0 (0)	0 (0)
Experienced, *n* (%)	4 (27)	5 (100)	14 (88)
Expert, *n* (%)	10 (67)	0 (0)	2 (12)

Themes are presented in accordance with the Health Belief Model, with particular emphasis on reactions that would impact on likelihood of taking recommended preventive actions.

### Understanding

Users found understanding the information presented by the risk calculator challenging, tending not to realise that the different formats were presenting the same information in different ways. As this male said, looking at the *Compare* format (Figure 8):

‘I find it difficult to get my head round this one, this representation’.

(II 102, 67 years, male)

Most users quickly identified their preferred format, but there was no unanimity about which format was easiest to understand. Framing was clearly important, with users preferring information presented positively. This participant was reacting to two different presentations of the same information — *Healthy Years* (Figure 2) and *Outlook* (Figure 4), namely that for people like her the average age of first heart attack or stroke would be 80 years:

II: ‘Now that doesn’t look quite so promising as the … as that diagram that said … that line that said I should survive to 80 odd without a heart attack or stroke. Now what this is saying, this is saying something completely different, this graph. This graph is saying, if I get to 80 odd, I’ve got, like a 50% chance of having a heart attack or stroke, well that is giving me completely different information to the … to the previous thing. I like … I like the previous diagram; that told me good stuff. This diagram, based on the same information, is telling me not so good stuff. I’m confused, I don’t get that. Which one … which one do I … well I guess I should work on the worst case scenario.

IV: What makes … why should you work on the worst case scenario?

IE: *Well, because I’m only human and I’ve got to die of something, you know, I’m not going to live forever. And 50/50 … 50/50 at age 80 is not the same as reading a graph that says I should get to 80 without one, do you see what I’m saying? That I’m getting conflicting information here.* (II 109, 55 years, female)

### Emotional reactions

Participants had strong emotional reactions to viewing personalised risk estimates, with different presentations eliciting different emotions. As this female put it comparing the *Balance* (Figure 7) and *Outcome* (Figure 6) formats:

*‘I felt more emotive when I saw the bodies lining up. Balance, was it? That’s the Balance one. I found that quite emotive. Little outcomes, they were … yes. Smiley faces doesn’t really make me feel serious about things’*.

(II 103, 69 years, female)

Most participants were surprised by their results, which often did not fit with their pre-existing beliefs. Unsurprisingly, where the calculator estimates were more optimistic than users’ pre-existing beliefs, they initially found this encouraging and cheering. Users whose pre-existing beliefs were more optimistic than the information presented by the calculator became worried and anxious:

‘So yes, I did feel quite encouraged that it wasn’t as dire as I’d thought, because to be honest, when I saw those levels, I thought to myself, oh, I’m looking at having complications in the next few years. But it won’t make me complacent, but it’s made me feel not so low.’

(II 119, 64 years, female)

‘From my point of view that’s useful to know that 77 is not good, because that’s quite a high probability, isn’t it? Yes, that’s pretty high chance, 77, of having a heart attack, that’s worrying; I wouldn’t want to have a heart attack by 77.’

(II 113, 59 years, male, viewing *Healthy Years* format, Figure 2)

### Acceptance that the risk information presented was personally relevant

Many participants tended to discount the validity and personal relevance of the information presented. This was particularly apparent when users had expected a better risk profile. Users had complex personal health beliefs, which incorporated family history, personal experience, and actions already taken to improve health. They used these to explain why the calculated risk or changes in risk were not relevant to them personally.

‘“Your heart age is about 65.” Well I don’t know whether I accept that, the machine tells me that so it must be on some data or something but I don’t know many other 65-year-olds who go walking with blokes who are 10 and 15 years younger and keep up with them. And I lead a very active life.’

(II 122, 65 years, male)

‘I think losing weight is more important than this. I don’t know. I think that’s got it wrong, yes.’

(II 103, 69 years, female)

### Relevance of selected outcomes

Many participants commented that they were not particularly concerned about having a heart attack or stroke. Participants tended to see these as treatable events, which could be survived without having too much adverse effect on quality of life. Many participants were much more concerned by the possibility of losing their sight, developing painful leg ulcers, or becoming disabled in some other way. Similarly, for many of the participants, the duration of their remaining life was less important than the quality of life. None of the participants reported viewing heart attack or stroke as a marker for other outcomes that may have been more personally relevant:

‘This was focused on heart attack and strokes it didn’t seem to be related to … But, certainly that would have been of interest, if I could reduce the risk of losing my sight and losing a limb, that would certainly be very motivating for me, you know. I mean, heart attack or stroke is not really on my agenda. Whether it’s on anybody else’s agenda, probably, I don’t know.’

(II 106, 72 years, male)

### Perceived benefits and barriers to action

Many participants were already aware of the importance of weight control and physical activity in staying healthy and tended to be comfortable altering estimates for weight, physical activity, or smoking. Altering HbA1c, blood pressure, or lipid levels was often challenging, however, as participants tended not to know what sort of levels they should be aiming for with these parameters, and often were uncertain about whether their own results were ‘good’ or not. To enable participants to see maximal benefits of change, if asked, the interviewer provided suggestions for a reasonable range of numbers to enter.

Different users reacted differently to changes in risk estimates; changes that seemed small to one person were viewed as highly significant by another. Some users were clearly motivated by the visual display linking outcomes to risk factors, such as this 49-year-old male:

‘When I started fiddling about with the blood pressure, reducing the blood pressure down from what it was down to what it is now, just shows that, yes, you can do stuff, what I’m doing is right, that’s fantastically encouraging and makes you feel really good’.

(II 108, 49 years, male)

Many users found the perceived benefit to be small, however, particularly in response to changes in weight or physical activity:

*‘No, that’s the one, that’s the one that I can change. If I change that* [exercise 4 hours a week] *to yes. 1.2 years. It’s quite a low return for quite a major effort it seems to me.’*

(II 106, 70 years, male)

In general, participants felt that healthy life gains of less than a year were irrelevant:

*‘I can have another year, that’s not much. I don’t consider that a major incentive, really’*.

(II 109, 55 years, female)

Some users found the whole exercise demotivating:

‘You just get the sort of hopelessness feeling where it doesn’t matter what you do, it’s going to slowly get worse and then it’ll kill you, which is what I hear all the time’.

(II 108, 49 years, male)

Linked to the concept of effort in achieving change was the concept of control. Whereas weight and physical activity were seen as under their control, many participants saw HbA1c, lipids, and blood pressure to be factors for which their doctor was responsible:

‘There’s three things to the treatment: the medication, the diet, and the exercise. And I’ve got control over two of those three things and I don’t believe the medication does much …’

(II 109, 55 years, female)

In contrast, some participants found the impact of changes in blood pressure or cholesterol highly motivating, and helped them overcome any antipathy to taking tablets:

‘I can do something about the blood pressure which is why I was very interested in it, I can take the tablets. Is it worth taking them? You’ve shown me this morning, yes, it is.’

(II 108, 49 years, male)

### Overall responses

Despite the expressed difficulties in understanding the outputs from the risk calculator and the widely varying emotional reactions to the data, overall, participants felt that the availability of such a tool was beneficial, and they wanted it made widely available. There was a widespread perception that access to this information would enable users to make informed choices and would be motivating. As this focus group user said:

‘And although it may be only a year’s difference, there were half a dozen things where you could make a year’s difference, and that starts, I mean you can see where your priorities should be. So the potential for this I think is great for diagnosis, for prognosis, for encouragement, all those things, and it’s just trying to help.’

(FG 2, male)

Almost all users would recommend it to a friend or other people with diabetes (particularly those who were seen as having room for improvement):

‘Oh yes, I think it’s the sort of thing that would be particularly useful to somebody who’s perhaps not behaving themselves, and are outside all the tolerances, and it might actually go to underline it’s not just Nurse Naggy sort of thing.’

(II 101, 68 years, male)

Many participants felt it would be best used with a nurse or doctor, however, who could help the user enter correct values, and explain the implications of the various outputs:

‘Well, basically, if it was within my power, I would say, you should go and see somebody who can go through this … But I’d push them to ask somebody.’

(II 118, 77 years, male)

## DISCUSSION

### Summary

In this qualitative study of people with type 2 diabetes, use of a personalised cardiovascular risk calculator elicited a range of cognitive and emotional reactions. The complexity and range of these reactions go a long way to explaining the apparent lack of impact of presenting personalised risk estimates to individuals in terms of changing modifiable risk factors. Despite the use of best practice in presenting risk, many users found the information confusing and hard to understand. The information triggered strong emotional reactions, and where the emotions triggered were negative or uncomfortable, this often led to discounting the information provided. Global cardiovascular risk was often not seen as a personally relevant outcome, and reductions in risk from behavioural modification or taking medication were not always motivating as the benefits were perceived as small. Despite this, users reported being pleased they had had the opportunity to use the calculator, and were keen that other people with diabetes should have similar opportunities, particularly if the calculator could be used with a health professional to help make sense of the outputs.

### Strengths and limitations

This study has many strengths, including combining different qualitative methods, including user testing and think-aloud interviews. These methods provided ‘real-time’ data and minimised the amount of self-censoring or social desirability bias. The intervention was clinically relevant to all the participants as their risk information was based on their own clinical data. Hence, participants were not being asked to consider their responses to a hypothetical situation. Data were analysed by a multidisciplinary team.

The major limitation to the data is that by definition, the participants were interested in self-management of their diabetes, as they responded to advertisements for people with diabetes to help develop and improve online self-management tools. Hence, many of the participants were already knowledgeable about diabetes, and felt that they had already taken considerable steps to improve their health. Despite this, many of the users were surprised to find they had average or above average levels of risk compared with other people with diabetes, suggesting that many of the study findings may transfer to patients less actively involved in self-management. Another consequence of the method of recruitment was that all except one of the participants described themselves as ‘experienced’ or ‘expert’ internet users. It is possible that less experienced users may have found the risk calculator even harder to understand and navigate than these relatively experienced users. Some patients had difficulty in obtaining their clinical data from their GP, and many found it hard to alter clinical parameters such as HbA1c or cholesterol, as they had little idea of appropriate ranges. This could be addressed in future versions of the calculator by providing guidance on appropriate ranges in the right-hand panel.

### Comparison with existing literature

The present study data support and build on previous literature in this field. The findings on the need for multiple formats for presentation of risk information agree with established best practice. The tendency to derogate or discount information that undermines an individual’s emotional wellbeing is also well documented,^11^,^26^ and there are techniques for minimising this effect, such as encouraging users to engage in a positive self-affirmation before accessing such information.^27^,^28^ It is hard, however, to see how these techniques could be routinely applied with a web-based risk calculator that users are free to use when and how they choose.

### Implications for research and practice

The study data suggest that, at present, cardiovascular risk calculators for people with diabetes may be best used in conjunction with health professionals who can guide the user through the calculator, ensure accurate data entry, and help use the resulting risk information as a source of motivation and encouragement. Consideration was made of removing the risk calculator from the HeLP-Diabetes intervention, but the users urged against doing this, therefore additional text was added with advice to use the calculator with clinical support. Further research is needed on how best to help users not only understand the information generated, but also to process it in a way that is constructive or helpful, so that initial anxiety or distress can be harnessed to help achieve change rather than result in demotivation or hopelessness. It is also worth acknowledging that benefits considered highly worthwhile at population or public health level may not be seen as significant by individuals. It is important to acknowledge the validity of this difference in perspective which may well lead to informed individuals making decisions about their health care or health behaviours at odds with current clinical guidance.^29^
